# Financial and Clinical Impact of Virtual Care During the COVID-19 Pandemic: Difference-in-Differences Analysis

**DOI:** 10.2196/44121

**Published:** 2023-01-25

**Authors:** Robert J Walter, Stephen D Schwab, Matt Wilkes, Daniel Yourk, Nicole Zahradka, Juliana Pugmire, Adam Wolfberg, Amanda Merritt, Joshua Boster, Kevin Loudermilk, Sean J Hipp, Michael J Morris

**Affiliations:** 1 Brooke Army Medical Center Joint Base San Antonio-Fort Sam Houston, TX United States; 2 Department of Economics Baylor University Waco, TX United States; 3 Current Health Ltd Edinburgh United Kingdom; 4 Current Health Inc Boston, MA United States

**Keywords:** remote patient monitoring, digital health, telemedicine, telehealth, finance, financial, cost, economic, remote care, readmission, cost savings, virtual care, patient care, digital health intervention, military health system, clinical outcome, military, soldier, difference-in-differences

## Abstract

**Background:**

Virtual care (VC) and remote patient monitoring programs were deployed widely during the COVID-19 pandemic. Deployments were heterogeneous and evolved as the pandemic progressed, complicating subsequent attempts to quantify their impact. The unique arrangement of the US Military Health System (MHS) enabled direct comparison between facilities that did and did not implement a standardized VC program. The VC program enrolled patients symptomatic for COVID-19 or at risk for severe disease. Patients’ vital signs were continuously monitored at home with a wearable device (Current Health). A central team monitored vital signs and conducted daily or twice-daily reviews (the nurse-to-patient ratio was 1:30).

**Objective:**

Our goal was to describe the operational model of a VC program for COVID-19, evaluate its financial impact, and detail its clinical outcomes.

**Methods:**

This was a retrospective difference-in-differences (DiD) evaluation that compared 8 military treatment facilities (MTFs) with and 39 MTFs without a VC program. Tricare Prime beneficiaries diagnosed with COVID-19 (Medicare Severity Diagnosis Related Group 177 or International Classification of Diseases–10 codes U07.1/07.2) who were eligible for care within the MHS and aged 21 years and or older between December 2020 and December 2021 were included. Primary outcomes were length of stay and associated cost savings; secondary outcomes were escalation to physical care from home, 30-day readmissions after VC discharge, adherence to the wearable, and alarms per patient-day.

**Results:**

A total of 1838 patients with COVID-19 were admitted to an MTF with a VC program of 3988 admitted to the MHS. Of these patients, 237 (13%) were enrolled in the VC program. The DiD analysis indicated that centers with the program had a 12% lower length of stay averaged across all COVID-19 patients, saving US $2047 per patient. The total cost of equipping, establishing, and staffing the VC program was estimated at US $3816 per day. Total net savings were estimated at US $2.3 million in the first year of the program across the MHS. The wearables were activated by 231 patients (97.5%) and were monitored through the Current Health platform for a total of 3474 (median 7.9, range 3.2-16.5) days. Wearable adherence was 85% (IQR 63%-94%). Patients triggered a median of 1.6 (IQR 0.7-5.2) vital sign alarms per patient per day; 203 (85.7%) were monitored at home and then directly discharged from VC; 27 (11.4%) were escalated to a physical hospital bed as part of their initial admission. There were no increases in 30-day readmissions or emergency department visits.

**Conclusions:**

Monitored patients were adherent to the wearable device and triggered a manageable number of alarms/day for the monitoring–team-to-patient ratio. Despite only enrolling 13% of COVID-19 patients at centers where it was available, the program offered substantial savings averaged across all patients in those centers without adversely affecting clinical outcomes.

## Introduction

### Background

The COVID-19 pandemic forced health care systems around the world to rapidly innovate and adapt to unprecedented operational and clinical strain [1]. Many health care systems leveraged virtual care (VC) capabilities to monitor patients while reducing staff exposure and managing resource constraints [2-4]. The US Department of Health and Human Services waived penalties for violations of the Health Insurance Portability and Accountability Act incurred through use of popular communication tools and the Centers for Medicare and Medicaid Services waived a number of regulations, paving the way for inpatient care in the home [5,6].

Early initiatives were heterogeneous, driven by local trends and available technologies [2]. They ranged from patients reporting single, manually recorded vital signs (such as pulse oximetry) to comprehensive packages of care delivered by dedicated clinical services and augmented by 24-hour continuous monitoring of multiple vital signs [7-9]. These deployments must now be evaluated and proven both clinically and financially effective for VC to continue, to justify the resources already invested in setting up these initiatives, and to better select suitable patients for care [6,10,11]. Some centers have reported reductions in length of hospital stay, intensive care admissions, and readmissions with VC [12,13]. However, evaluation has been complicated by the variation in approaches to VC in different institutions, which evolved in parallel with new COVID-19 treatments, vaccinations, and viral variants, and by selection bias between monitored and unmonitored cohorts [2,13,14]. A related concern has been the “digital divide,” the variation in access to digital technologies across age, educational, socioeconomic, and geographical strata, limiting the reach of VC services [15,16].

In December 2020, the US Military Health System (MHS) Virtual Medical Center (VMC) implemented a VC program in 8 military treatment facilities (MTFs) across the United States for COVID-19 patients. The program was delivered virtually by a 24-hour dedicated nursing service using continuous remote patient monitoring. The implementation of a standardized VC program for the same condition in a subset of similar treatment facilities across the country made for an ideal natural experiment.

### The VC Program

The VC program was available to any Tricare Prime beneficiary eligible for care within the MHS aged 21 years or older. Inclusion criteria were broad and included any patient who presented to hospital symptomatic for COVID-19 and those who, despite not requiring admission, had a high risk of exposure and were at risk for severe disease due to a comorbid state. The program was subsequently expanded to other use cases, including congestive heart failure, gestational hypertension, and postoperative monitoring for bariatric surgery; however, this analysis only pertains to COVID-19.

Referred patients were screened for eligibility and consented to participation in the VC program, and they were then issued the VC equipment and familiarized with it by specially trained nurses. Once home, patients were called to ensure kit setup was successful. Enrollment triage forms (Multimedia Appendix 1) stratified patients as low, medium, or high risk, dictating the frequency of patient engagement. Low-risk patients were monitored intermittently, medium-risk patients were reviewed daily, and high-risk patients were reviewed twice daily. All reviews were conducted virtually.

Patients were monitored using the Current Health (CH) VC platform (Current Health Inc), which included a US Food and Drug Administration (FDA) 510(k)-cleared wearable device worn on the upper arm, a blood pressure cuff and weighing scale, a tablet, and a network hub that operated via home Wi-Fi or roaming cellular signal, enabling access for patients without home internet. Continuous vital signs measured were pulse rate, respiratory rate, oxygen saturation, temperature, and motion. Blood pressure and weight were monitored intermittently. The tablet collected customizable patient-reported outcome measures, including symptom burden, and could be used to asynchronously request direct assistance from the on-call nursing team. All data were processed via cloud computing and displayed on a web dashboard for the clinical team. If any vital sign exceeded a predetermined threshold, an alert would trigger on the dashboard and send notifications to the appropriate staff. The team monitored patients across the 8 participating MTFs with a nurse-to-patient ratio of 1:30. Each MTF had designated on-call physicians available for on-demand support if care escalation was required.

Patients were disenrolled from VC at their physicians’ discretion or if escalated back from VC to inpatient care due to clinical decompensation. CH coordinated kit return, sanitization, service, and repackaging and returned the kits to their original MTF. This ensured that each MTF maintained a consistent stock of equipment to enroll new patients. The infrastructure, personnel, and fiscal resources for the program were directly funded by the MHS. A total of 200 CH kits were available for distribution across the 8 participating MTFs, dynamically divided based on utilization and demand. There were always sufficient kits available at each location.

## Methods

### Econometric Analysis

The difference-in-differences (DiD) model used ordinary least squares regression, regressing the outcome on an indicator for whether the hospital was included in the VC program, an indicator for whether the patient’s date of admission was after the earliest implementation of the VC program, and an interaction variable of the 2 indicators. The coefficient of this interaction can be interpreted as the effect of being admitted to a hospital that had an active VC program regardless of enrollment in the program. The model controlled for age, gender, marital status, COVID-19 pneumonia diagnosis during the index admission, and Elixhauser comorbidity score [17]. Fixed effects were included for the hospital and the quarter year. The final estimate was the average within-hospital change in length of stay in hospitals that implemented a VC program compared to hospitals that did not. To understand which patients were most affected by the program, a predicted length of stay was estimated based on observable characteristics of the patients. This estimate was computed in two stages. In the first stage, the observed length of stay was regressed on patient age (linearly and quadratically), gender, marital status, Elixhauser comorbidity score, and an indicator for whether there was a pneumonia diagnosis. This produced a partial correlation between each of these covariates and length of stay. These partial correlations were then applied to each patient’s observed covariates to generate a predicted length of stay for each individual. The residual length of stay was then calculated as the difference between the predicted and actual lengths of stay. This residual can be interpreted as the portion of the stay that was not attributable to observed characteristics of the patient.

The sample was constructed using all Tricare beneficiaries admitted to an MTF with COVID-19 from December 7, 2020, to December 6, 2021. Only the patient’s first admission was included, with patients admitted either directly by their physician or through an emergency department. Patients with any Medicare Severity Diagnosis Related Groups other than 177 were excluded, along with any who were not discharged to their home. The final sample included 3988 index admissions: 1838 patients who were admitted into an MTF with an VC program and 2150 who were admitted to an MTF without a program. Of the 1838 patients admitted into an MTF with a VC program, 237 (13%) were enrolled in VC. The average cost of VC was calculated per day based on the capital expenditure and ongoing monitoring contract, costs of nursing labor, and program management support. While VC program initiation varied at the MTF level, the Defense Health Agency (DHA) paid for the VC centrally with a single, centralized monitoring hub. This makes sense given that VC is a high fixed-cost investment that can be managed from a single location, allowing for economies of scale. However, this means that costs can only be calculated at the system level and not at the MTF level.

### Clinical Outcomes for the Cohort Enrolled in the VC Program

Outcome data for the 237 patients enrolled in the program were obtained from MHS Mart, known as “M2,” which is a queryable data repository for the DHA. Vital-sign and alarm data were obtained from CH. A manual chart review of patients’ electronic medical records (EMRs) was conducted for the subgroup of patients at Brooke Army Medical Center (BAMC) between December 7, 2020, and June 7, 2021. The review was limited to the first 6 months of the program due to the availability of clinicians to conduct the review. That review focused on comorbidities that increased risk for severe COVID-19, including smoking, diabetes, being immunocompromised, chronic kidney disease, and hypertension, as well as validated scores for severity and readmission: the Quick COVID-19 Severity Index (qCSI), Quick Sepsis Related Organ Failure Assessment (qSOFA), and the HOSPITAL score (an abbreviation that represents “hemoglobin at discharge, discharge from an oncology service, sodium level at discharge, procedure during the index admission, index type of admission, number of admissions during the last 12 months, and length of stay”) [18-20]. The manual review was given precedence if there was a conflict between the M2 database and the EMR reviews. Data were analyzed in R (R Foundation for Statistical Computing). Results were quantified, assessed for normality (via visualization with the Shapiro–Wilk test), and presented as the mean (SD) or, if nonparametric, the median (IQR). Wearable adherence was defined as hours of data transmitted divided by the time between the first and last transmission. Differences between groups were assessed using the Wilcoxon rank sum test or chi-square test (without Yates correction), as were nonparametric data, with significance at *P*<.05. Multiple comparisons were corrected using the Holm–Bonferroni method.

### Ethics Approval

The study complied with the Declaration of Helsinki. This was a retrospective study of data collected for clinical rather than research purposes, so prior informed consent was not sought and no compensation was offered. An exemption for retrospective analysis was granted by the BAMC Institutional Review Board (reference number C.2021.103e). The data were deidentified for analysis.

## Results

### Econometric Analysis

During the study period, 3988 patients were admitted to the MHS with COVID-19. Table 1 compares patient covariates for those seen at MTFs with and without VC. Patients were similar in age, Elixhauser index, and rates of pneumonia listed on index admission. There was a trend toward more female patients at hospitals with VC (*P*=.08), but this result was not statistically significant. The patients were significantly more likely to be married. A strict balance on these covariates was not required for the DiD because the changes in the distribution did not coincide with the timing of the program; the marginal difference in genders disappeared when looking at the postimplementation period, while the marital difference remained constant [21,22].

**Figure 1:**
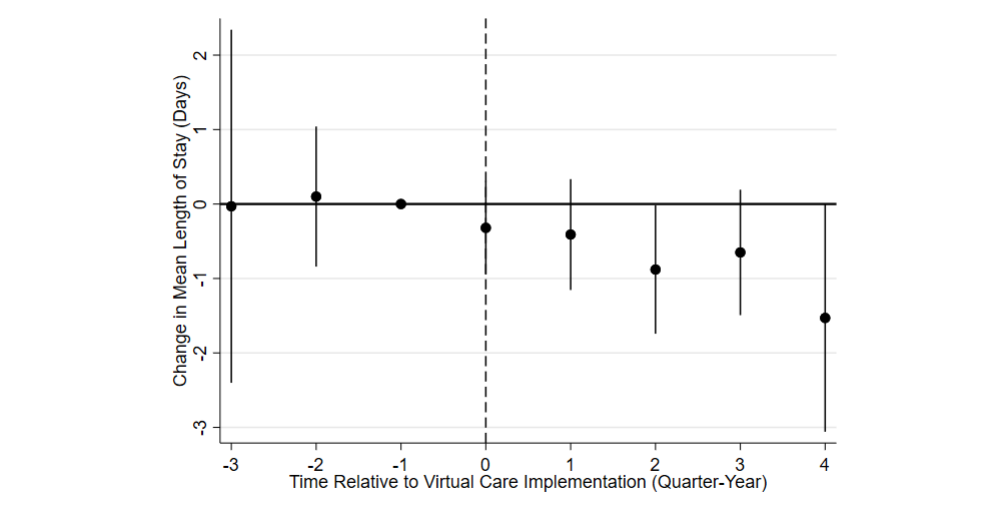
Difference in adjusted means of the treatment and control military treatment facilities before and after implementation of the virtual care program.

The first column in Table 2 displays the results of the DiD analysis, and Figure 1 shows the difference in adjusted means of the treatment and control hospitals before and after implementation of VC. VC was associated with a 0.56-day (12%, *P*<.031) reduction in length of stay for all COVID-19 patients at an MTF, with no increase in readmissions or emergency department visits. The average Tricare Medicare Severity Diagnosis Related Group (DRG) payment, excluding graduate medical education and other add-ons for DRG 177, was US $16,568. Tricare calculated the per diem payment (used on hospital transfers) as the total payment divided by the geometric mean length of stay for that DRG. Using this methodology each day in the hospital cost US $3682. This implied savings of US $2047 per patient for every COVID-19 patient admitted to a center with VC available, or US $3,762,386 in total. At the hospital level, the total cost of equipping, establishing, and staffing the VC program was estimated at US $3816 per day, or US $1,392,840 per year. Total net savings were therefore estimated at US $2.3 million in the first year of the program across the MHS.

**Figure 2:**
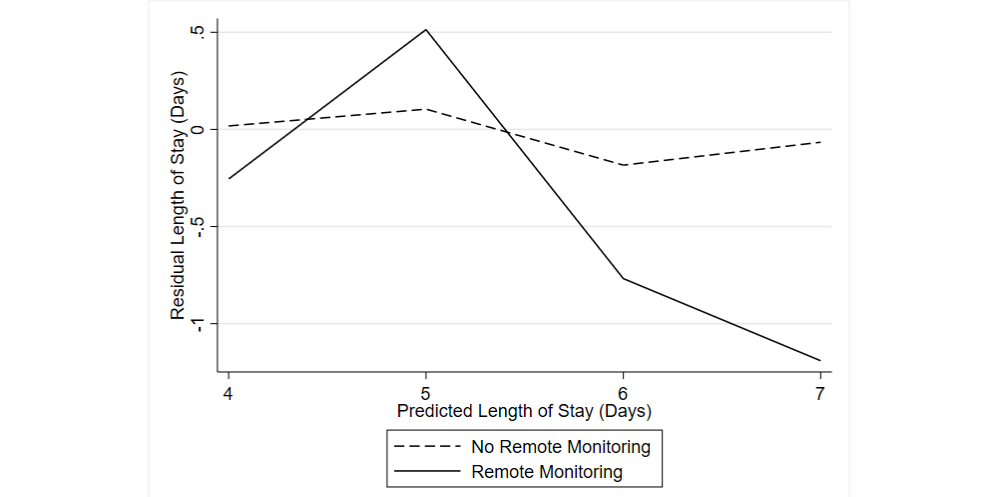
Residual versus predicted length of stay for those on virtual care, based on observable covariates and actual length of stay.

The DiD provided an average effect, but conceptually the program should have impacted those that would have stayed in the hospital for monitoring. Figure 2 displays plots of the residual length of stay for patients in the VC and non-VC groups. The program appears to have reduced length of stay most effectively for those that would have otherwise been in the hospital longest.

**Table 1:** Patient covariates at military treatment facilities with a virtual care program (treatment), and without a program (control) (N=3988).

Characteristics	Treatment (n=1838)	Control (n=2150)	% Difference (treatment minus control)	*P* Value
Mean age (years)	55.98	56.27	–0.29	.58
Female, n (%)	717 (39)	774 (36)	3	.08
Married, n (%)	1489 (81)	1613 (75)	6	<.001
Pneumonia, n (%)	1011 (55)	1226 (57)	–2	.21
Mean Elixhauser score	1.96	1.98	–0.02	.78

**Table 2:** Results of difference-in-differences analysis (N=3988). All regressions include the full set of control variables and fixed effects.

	Length of stay (n=3984)	Thirty-day readmission (n=3984)	Emergency department visit within 90 days (n=3984)
Panel A: linear (postimplementation), coefficient (SE)	–0.556 (0.256)	0.007 (0.019)	–0.011 (0.018)
Panel B: log transformation (postimplementation), coefficient (SE)	–0.135 (0.047)	N/A^a^	N/A
Panel C: alternative specification (postimplementation), coefficient (SE)	–0.115 (0.051)^b^	0.094 (0.249)^c^	0.45 (0.501)^c^
Full sample mean	4.78	0.08	0.02

^a^N/A: not applicable.

^b^Alternative specification: Poisson.

^c^Alternative specification: logit.

### Clinical Outcomes for the Cohort Enrolled in the VC Program

For the 237 patients enrolled in the program, mean age was 53 (SD 15.3) years, 100 (42%) were female, 137 (58%) were male, 231 (97.5%) activated their wearable, median activation time was 60 (IQR 11-186) minutes, and they were monitored through the CH platform for a total of 3474 (median 7.9, IQR 3.2-16.5, range 1-106) days. Wearable adherence was 85% (IQR 63%-94%). Patients triggered a median of 1.6 (IQR 0.7-5.2) physiological alarms per patient per day; 203 (85.7%) were monitored at home and directly discharged from VC; 27 (11.4%) were escalated to a physical hospital bed while on monitoring; and 1 (0.4%) was readmitted to the hospital within 30 days of discharge from VC. There were no deaths in the cohort. There were significant differences between those requiring escalation to physical care and those remaining at home throughout their time in the VC program. Those who required escalation activated their CH kit in a similar timeframe but were less adherent to using the wearable (median 63%, IQR 32%-83% vs 87%, IQR 70%-95%, respectively; *P*<.001), although they generated significantly more physiological alarms per 24 hours of monitoring (median 7.0, IQR 1.9-17.3 alarms vs median 1.3, IQR 0.6-3.6 alarms; *P*<.001) despite the decreased wear time.

Charts from 39 patients (16% of the monitored cohort) from the first 6 months of the VC program at BAMC were hand-reviewed for COVID-19 risk factors. Their demographics and COVID-19 risk factors are presented in Table 3. The patients in the subset were monitored for a total of 684 (median 8.8, IQR 3-12, range 1-45) days. Thirty-eight (97.4%) activated their wearable for a median 34 (IQR 1-125) minutes, and wearable adherence was 78% (IQR 60%-91%). Patients triggered 1.7 (IQR 0.8-5.6) physiological alarms per day. Thirty-four (87%) of the 38 remained at home during their monitoring period and 4 (10.3%) were escalated to physical care during their initial admission. Once discharged from hospital and VC, there were no readmissions in the subsequent 30 days (the subset’s HOSPITAL scores would have predicted a 30-day readmission rate of 5.8%, equivalent to 2 or 3 of the 39 patients reviewed).

**Table 3:** Demographics and COVID risk factors of patients enrolled in the virtual care program at Brooke Army Medical Center (n=39).

Characteristics		Values
Age (years), mean (SD, range)		59 (14.6, 31-86)
**Sex, n (%)**		
	Female	15 (38)
	Male	24 (62)
**Ethnicity, n (%)**		
	Asian	1 (3)
	Black	7 (18)
	Hispanic	12 (31)
	Other or unspecified	3 (8)
	Southeast Asian	1 (3)
	White	15 (38)
**Triage stratification, n (%)**		
	High risk	25 (64)
	Medium risk	14 (36)
**COVID-19 risk factors**		
	Smoker, n (%)	6 (15)
	Diabetes, n (%)	12 (31)
	Immunocompromised, n (%)	11 (28)
	Chronic kidney disease, n (%)	4 (10)
	Hypertension, n (%)	23 (59)
	Risk factors, median, (IQR, range)	1 (0-2, 0-4)
**Quick Sepsis Related Organ Failure Assessment score groups, n (%)**		
	0	21 (54)
	1	17 (43)
	2	1 (3)
HOSPITAL^a^ score, median (IQR, range)		2 (1-2.5, 0-6)
**Intensive care unit admission while in hospital, n (%)**		
	No	37 (95)
	Yes	2 (5)
**Discharged on oxygen, n (%)**		
	No	13 (33)
	Yes	26 (67)

^a^HOSPITAL score: hemoglobin at discharge, discharge from an oncology service, sodium level at discharge, procedure during the index admission, index type of admission, number of admissions during the last 12 months, and length of stay.

## Discussion

The MHS VC program was established during a time of acute national need, with patients offered round-the-clock remote care as an alternative to being in the hospital. Despite only enrolling 6% of patients with COVID-19 admitted to the MHS and only 13% of patients at centers where the program was available, the program had a disproportionately large impact. Overall length of stay was reduced by 12%, averaged across all COVID-19 patients at centers with availability, with an associated cost saving of $2047 per patient. Reassuringly, the 11% rate of escalation to physical care for patients enrolled in the program demonstrated that unwell patients were being identified and treated despite being at home, with no increase in emergency department attendance, 30-day readmissions, or deaths. It should be stressed that escalation to physical care was not a “readmission,” as the patient remained in their “initial admission” until discharged from the program. Indeed, escalation was desirable in those patients who warranted it, and any days spent at home rather than in the hospital increased inpatient capacity at the facilities while reducing exposure to COVID-19 for other patients and staff.

The median length of monitoring on VC was 7.9 days, and the overall adherence to wear was 85%. Adherence to wearables has typically been reported as the total number of days patients wore a device, rather than the consistency of wear during those days. In both postoperative and clinical trial contexts, median length of engagement has been reported at around 5 days [23,24]. It was notable that patients less adherent to the wearable were more likely to require escalation to physical care. Lower adherence may have reduced health care provider confidence in the patient staying at home, precipitating admission, or led to deteriorations being caught later, after simple steps such as increasing fluid intake were no longer effective. Alternatively, those who were more unwell may have been less inclined to wear the wearable.

Other previously identified barriers to VC adoption have included lack of connectivity (the so-called digital divide that disproportionately affects the elderly, those with low income, and rural populations) and concerns around privacy and usability [4,25,26]. The CH platform included a roaming cellular function, so the patients did not require their own internet connection. The data were transmitted from the patient’s home as raw waveforms and did not include any patient identifiable information. The VMC team were able to address usability concerns when handing over the kit in hospital and following up by phone.

The alarms needed to be specific as well as sensitive to avoid disturbing patients, bringing them into the hospital unnecessarily, or increasing the risk of alarm fatigue among the nursing team [27]. There is a significant relationship between alarm exposure and response time [28]. It has previously been reported that actionable alarms are already a low percentage (20%-36%) of the total numbers of alarms triggered in adult ward settings [28]. Alarm actions were not tracked directly, but the alarm rate of 1.6 per patient-day was manageable in the context of a 1:30 nurse-to-patient staffing ratio and is lower than previously reported in-hospital and at-home alarm rates of 10.8 per patient-day and 3.42 per patient-day, respectively [29,30].

The facilitators and barriers to rolling out this intervention were the subject of a separate qualitative study, presently under review. However, to contextualize the findings of this paper, we noted that in common with similar interventions, clinician acceptance took time to establish, and more patients would have likely been enrolled if acceptance had come sooner [31,32]. The program also benefited in some ways from its military setting. Nurses, licensed and credentialed at a single MTF, could practice across the whole health system. That, along with the clear leadership structures and hospital physicians being employed by a single entity, facilitated rapid expansion across the country. The unique medical malpractice conditions of the MHS, where legal action cannot be taken against individual providers, may have made the clinicians more willing to take responsibility for patients’ remote care.

However, the program was also hampered by the inability to coordinate community services or go into patients’ homes. The lack of an integrated inpatient/outpatient EMR, along with inexperience in the use of virtual care relative value units and Current Procedural Terminology–associated reimbursement also slowed revenue generation. Finally, the CH platform was FDA 510(k)-cleared only for patients older than 21 years, which made the proportion of the active-duty population aged between 18 and 21 ineligible for enrollment.

The unique strength of this study was its comparison of similar health facilities spread across the United States all attempting to treat an identical clinical condition concurrently. The DiD analysis compared a treatment group (centers with VC) to a control group (centers without VC) before and after the intervention (ie, VC), then estimated the divergence in outcome (ie, length of stay). The identifying assumption was that the treatment group would have followed parallel trends with the control group in the absence of the intervention. In other words, changes in the dominant COVID-19 variant, vaccination rates, treatment methodologies, or other factors would have impacted centers with and without VC equally. While this was an inherently untestable assumption, Figure 1 plots the difference in adjusted means of the treatment and control MTFs before and after implementation of the VC program, demonstrating parallel pretrends (ie, both the treatment and the control groups followed the same trajectory prior to the initiation of the VC program). DiD methodology also requires quasi-random (ie, uncorrelated with the intervention) assignment to the treatment. MTFs were not randomized to set up VC programs, but the hospital that someone attended could be considered quasi-random (patients with COVID-19 were unlikely to travel to a hospital on the grounds that it had a VC program). The Elixhauser comorbidity index indicated that patients included in the analysis were similar at MTFs with and without VC. However, enrollment in VC could not be considered random, as patients were selected.

Consequently, the DiD analysis could only estimate the effect that the presence of VC had on patients on average. It demonstrated that patients at centers with VC stayed in the hospital for less time than their counterparts in centers without VC. This may have been due to the specific patients being entered in the program but could also have been driven by hospitals and physicians having more ability to focus on those patients who remained in the hospital. The VC effect may also have been driven through creating additional capacity, as well as the care itself. To this point, Figure 2 suggests that the reduction in length of stay came from individuals who would otherwise have spent a long time in the hospital being able to go home with VC. However, given the limitations above, the separate contributions of patient selection, nursing care, logistics, and technology could not be easily parsed.

In conclusion, the unique structure of the MHS allowed comparison between MTFs that implemented a VC program for COVID-19 and those that did not. Despite the VC program enrolling only a small proportion of patients admitted with COVID-19, it offered substantial savings in centers where it was adopted. The program was effective in identifying suitable patients, escalating them appropriately to physical care, and discharging them once their illness was resolved. The program's military context may have aided its rapid rollout and adoption across multiple centers, and the single-payer nature of the DHA may have facilitated the economic justification of the initiative. However, the results are likely to be applicable to other large health systems that can support or engage a nurse monitoring service, particularly those systems that can reap the economic benefit of a cost-saving program.

## Appendix

Multimedia Appendix 1 Enrollment form and risk stratification for the Virtual Care program.

## Abbreviations

BAMC: Brooke Army Medical Center
CH: Current Health
DHA: Defense Health Agency
DiD: difference in differences
DRG: Diagnosis Related Group
EMR: electronic medical record
FDA: US Food and Drug Administration
HOSPITAL score: hemoglobin at discharge, discharge from an oncology service, sodium level at discharge, procedure during the index admission, index type of admission, number of admissions during the last 12 months, and length of stay
M2: Military Health System Mart
MHS: Military Health System
MTF: military treatment facility
qCSI: Quick COVID-19 Severity Index
qSOFA: Quick Sepsis Related Organ Failure Assessment
VC: virtual care
VMC: Virtual Medical Center

## Data Availability

The data sets generated during and/or analyzed during the current study are not publicly available due to information governance restrictions within the Military Health System. Please make any requests for access to MJM (michael.j.morris34.civ@health.mil), who is chairman of the Brooke Army Medical Center Institutional Review Board.
