# Supplementary material for: Financial and Clinical Impact of Virtual Care During the COVID-19 Pandemic: Difference-in-Differences Analysis
Source: J Med Internet Res. 2023 Jan 25;25:e44121. doi: 10.2196/44121 (PMC9879318; doi:10.2196/44121)
Supplement: Multimedia Appendix 1 [file jmir_v25i1e44121_app1.pdf]

COVID-19 Remote Patient Monitoring (CRPM) Enrollment Form

Referring provider to complete this section	**Date:** (YYYY-MM-DD)

**Patient Name (Last Name, First Name, MI):**	**DoDID:**	**DOB/Age:** / years
**Diagnosis:**	**COVID-19 Status:** ☐ Positive ☐ Negative ☐ Pending ☐ N/A	**Admitting Physician:**

Please Select :

**High Risk:** ________	**Medium Risk:** ________	**Low Risk:** ________
High Risk: Resting or exertional O2 requirement upon discharge; or high-risk co-morbid conditions (e.g.: HF, CLD, PHTN)	Medium Risk: Resting or exertional dyspnea upon discharge; or moderate-risk co-morbid conditions (e.g.: DM2, HTN, obesity)	Low Risk: No significant resting or exertional dyspnea; or no significant co-morbid conditions
Monitoring: Continuous, passive vital signs +/- integrated peripherals	Monitoring**:** Continuous, passive vital signs +/- integrated peripherals	Monitoring: Intermittent vital signs
Engagement: Twice daily review (or by alert) of clinical status, weekly video call	Engagement**:** Once daily review (or by alert) of clinical status, weekly video call	Engagement: As needed by alert

Monitored VS Basic Parameters:

Abnormal VS:
- **Temperature: ≥ 101° F or ≤ 96.5° F**
- **Respiratory Rate: ≥ 20 or ≤ 8**
- **Heart Rate: ≥ 120 or ≤ 40**
- **SpO2: ≤ 88%**

Extremely Abnormal VS:
- **Temperature: ≥ 103° F or ≤ 95° F**
- **Respiratory Rate: ≥ 30 or ≤ 5**
- **Heart Rate: ≥ 150 or ≤ 30**
- **SpO2: ≤ 85%**

Please indicate alternate parameters if desired:

Will CPRM Enrollment Prevent an In-Patient Admission? ☐ YES ☐ NO ☐ N/A

Will CPRM Enrollment Decrease In-Patient Bed-Days? ☐ YES ☐ NO ☐ N/A **If yes, approx. how many? _____**

Comments: (Pertinent History of Present Illness)

**Provider Signature:**	**Print Name:**	**Phone Number:**

Nursing/Support staff to complete remainder of form

Referring MTF:

Sending/Admitting Department:

Anticipated Time/Date of D/C:

Gender:
☐Male ☐Female

Ht: _______

Wt: _______

Preferred language: *Language chosen will be language patient will see on tablet.*
☐English ☐Spanish

Phone Number: ☐Cell ☐Home

Email Address:

County:

Physical Address (NOT mailing address):

City:

Zip:

Ethnicity:
☐ White (Not Hispanic or Latino)
☐ Native Hawaiian or Other Pacific Islander (Not Hispanic or Latino)
☐American Indian or Native Alaskan (Not Hispanic or Latino)
☐ Asian (Not Hispanic or Latino)
☐ Hispanic or Latino
☐ Black or African American (Not Hispanic or Latino)

Any pacemaker or other implants? Indicate type/place
☐Yes ☐No ______________________
**Do not charge device while wearing*

Any upper arm sleeve tattoos?
☐Yes ☐No ☐Right ☐Left
**Patients with bilateral upper arm tattoos are ineligible to wear device*

Alternate/Emergency Contact Person & Relationship:

Phone number: **(Different from above)**

CORE KIT ID: *(ex: green uat nine)*

MULTI KIT ID: *(ex: cyan uat five)*

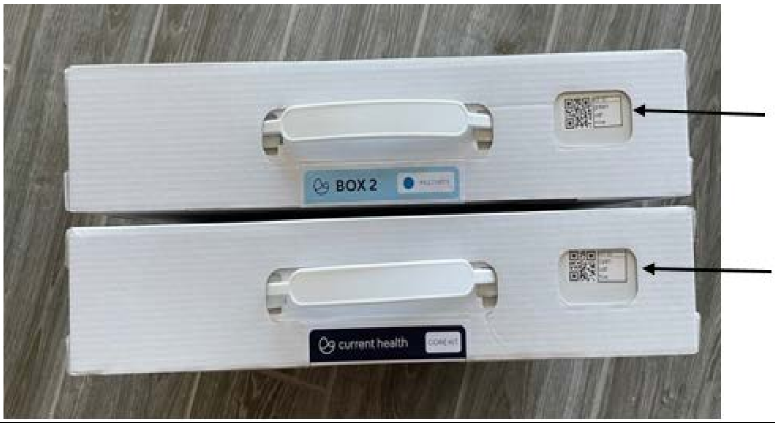

MULTI-KIT ID

CORE KIT ID

Additional Comments: (Home O2, pain, etc.)

Inpatient Unit Name:

Nurse's Station Telephone Number:

Prepared by: (Name, Signature, Title)

Department:

Contact Number:

Time/Date Prepared:

Please upload this complete form and the Informed Consent into the **Virtual Health Care Coordination Application (VHCCA)** site in CarePoint at the following link:
